# False positives in trans-eQTL and co-expression analyses arising from RNA-sequencing alignment errors

**DOI:** 10.12688/f1000research.17145.2

**Published:** 2019-04-08

**Authors:** Ashis Saha, Alexis Battle

**Affiliations:** 1 Department of Computer Science, Johns Hopkins University, Baltimore, Maryland, 21218, USA; 2 Department of Biomedical Engineering, Johns Hopkins University, Baltimore, Maryland, 21218, USA

**Keywords:** Mappability, Cross-mappability, Co-expression, Trans-eQTL, RNA-sequencing, Alignment

## Abstract

Sequence similarity among distinct genomic regions can lead to errors in alignment of short reads from next-generation sequencing. While this is well known, the downstream consequences of misalignment have not been fully characterized.  We assessed the potential for incorrect alignment of RNA-sequencing reads to cause false positives in both gene expression quantitative trait locus (eQTL) and co-expression analyses. Trans-eQTLs identified from human RNA-sequencing studies appeared to be particularly affected by this phenomenon, even when only uniquely aligned reads are considered. Over 75% of trans-eQTLs using a standard pipeline occurred between regions of sequence similarity and therefore could be due to alignment errors. Further, associations due to mapping errors are likely to misleadingly replicate between studies. To help address this problem, we quantified the potential for "cross-mapping'' to occur between every pair of annotated genes in the human genome. Such cross-mapping data can be used to filter or flag potential false positives in both trans-eQTL and co-expression analyses. Such filtering substantially alters the detection of significant associations and can have an impact on the assessment of false discovery rate, functional enrichment, and replication for RNA-sequencing association studies.

## Introduction

Sequence similarity among distinct genomic regions makes alignment of short sequencing reads difficult ^1, 2^. Genomes, including the human genome, contain diverse classes of elements with sequence similarity across regions, ranging from large segmental duplications to pseudogenes to transposable elements. Alignment-based quantification of genomic phenotypes such as gene expression or epigenetic signal is less reliable for such regions ^3– 6^.

**Figure 1.**
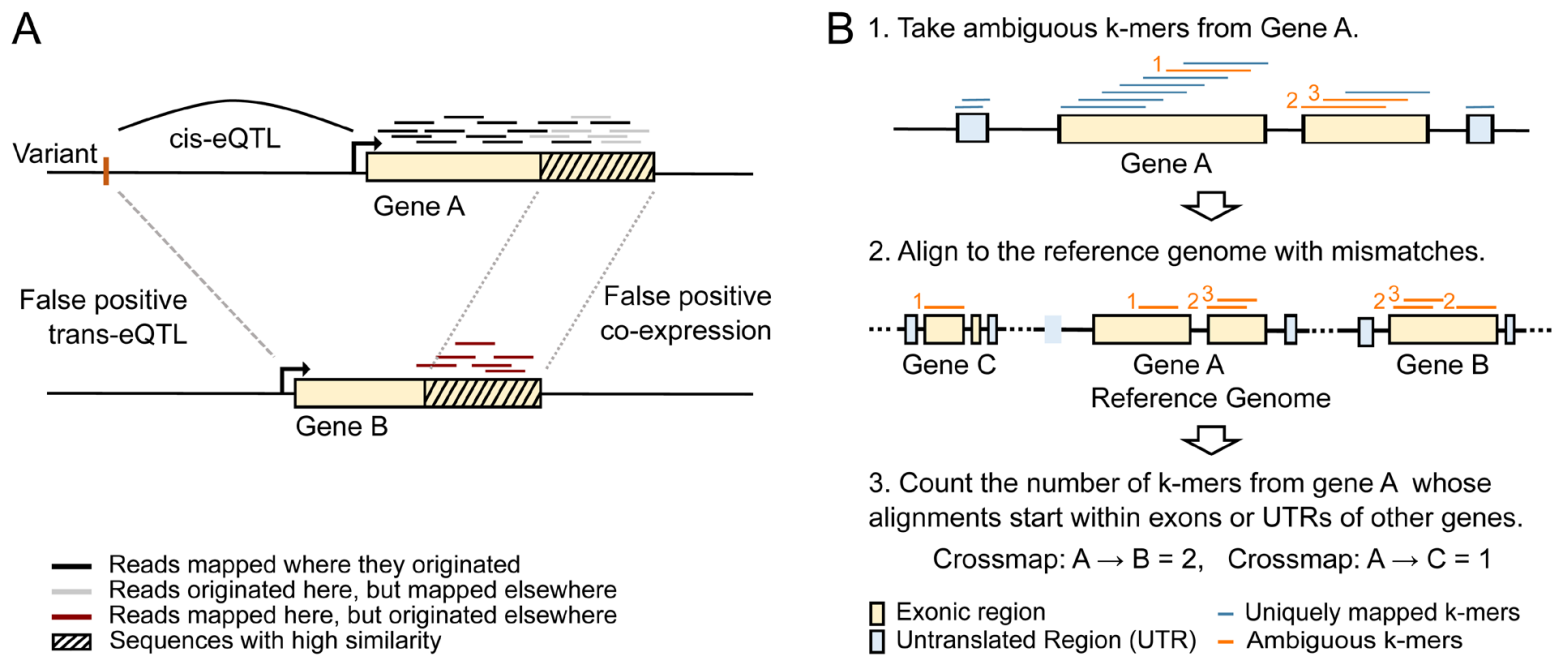
Overview of cross-mappability. **A**) Some of the reads generated from Gene A are incorrectly mapped to Gene B because of sequence similarity between the genes, leading to false positive co-expression. Consequently, a variant which is a true cis-eQTL of Gene A appears as a false positive trans-eQTL of Gene B. **B**) We align the ambiguous (orange) *k*-mers (75-mers from exons and 36-mers from UTRs) from Gene A to the reference genome using Bowtie and count how many *k*-mers from Gene A map to each other gene to compute cross-mappability. Here, the number beside each ambiguous (orange) *k*-mer represents the identifier for the ambiguous *k*-mer based on its position in Gene A.

Despite attention to the importance of alignment errors, the full range of consequences is not always considered in downstream analyses. Here, we focus on evidence that sequence similarity between pairs of genes and resulting alignment errors between them may lead to false positives in association studies from RNA-sequencing (RNA-seq) data, specifically in expression quantitative trait locus (eQTL) and co-expression analyses. eQTL studies, revealing associations between genetic variants and gene expression levels, have contributed to a greater understanding of gene regulation and genetics of complex traits ^7– 9^. Trans-eQTLs, where the genetic variant is distant or on a different chromosome from the associated gene, are of particular interest, but have proven challenging to identify in human data due to power, confounders, small effect sizes, and other challenges ^10, 11^. Given that a trans-eQTL analysis performs genome-wide tests, it is more prone to be affected by systematic errors between genomic regions than a cis-eQTL analysis where only variants close to the target gene are considered. Here, we discuss the impact of alignment errors on RNA-seq association studies. Figure 1A illustrates a cartoon example, where all reads truly originate from transcripts of Gene A, but due to sequence similarity between Gene A and Gene B, some of the reads incorrectly map to Gene B, causing it to erroneously appear to be expressed in the sample. The number of reads misaligned to Gene B across samples may be directly proportional to the number of reads for Gene A, or may be determined by genetic variation creating sequence mismatches with the correct region. In either case, spurious associations can then arise. In Figure 1A, the two genes incorrectly appear to be co-expressed. In addition, a variant associated with expression of Gene A may also appear to be associated with Gene B, giving rise of a false positive trans-eQTL. We note that such errors are not entirely mitigated by filtering multi-mapped reads—some alignment errors may remain between similar regions even among uniquely aligned reads due to genetic variation, errors in the reference genome, and other complications.

Previous studies have shown that uniqueness of sequence in genomic regions should be considered in an analysis of sequencing data ^4, 5, 12^. Karimzadeh *et al.* showed that a differential methylation analysis can identify false signals due to poor mappability ^5^. We have previously filtered trans-eQTLs based on sequence similarity as part of the Genotype-Tissue Expression (GTEx) project ^10^ and the Depression Genes and Networks (DGN) study ^13^. Pickrell *et al.* ^14^ also suggested that the most significant distant eQTL in their RNA-seq study was likely an artifact arising due to sequencing reads originating from a gene near the SNP mapping to another distant gene. Related effects were also discussed in greater depth for microarrays, where probes intended for one gene may cross-hybridize to other genes ^11, 15^. In microarray studies, one could identify and replace probes displaying poor specificity, but in RNA-seq, any region of sequence similarity between genes can cause alignment errors. Previous studies have not presented a systematic analysis of alignment-related false positives in RNA-seq association testing.

Here, we report the prevalence of potential false positives in trans-eQTL and co-expression analyses arising from alignment errors. We present a method to assess the potential for mapping error between pairs of genes, which can then be used to filter or flag associations that could arise from these errors. We introduce a new metric, “cross-mappability”, representing the extent to which reads from one gene may be mapped to another gene. Using gene expression data from GTEx ^10^ and DGN ^13^, we demonstrate the impact of misalignment on both trans-eQTL detection and co-expression analysis in real data. Notably, we show that over 75% of trans-eQTLs detected in any GTEx tissue using a naive pipeline are potential false positives, emphasizing that it is critical to consider these errors. To support future studies, we have published codes in Github ^16^ and also made cross-mappability resources publicly available for the human genome (hg19 and GRCh38) ^17^.

## Methods

### Mappability and cross-mappability

We developed a new metric, cross-mappability, to quantify the potential for incorrect read alignment where reads originating from one gene may incorrectly map to another gene. Based on annotated transcripts for each gene, we evaluated *k*-mers from exonic and untranslated regions (UTRs) of the reference genome that serve as a proxy for reads in an RNA-seq experiment. We defined cross-mappability from Gene A to Gene B, crossmap(A, B), as the number of Gene A’s *k*-mers whose alignment, allowing mismatches, start within exonic or untranslated regions of Gene B. Notably, existing *mappability* scores ^4, 5^ correspond to a single region (or gene) describing uniqueness of the sequence of the region in the genome, our *cross-mappability* score corresponds to a pair of genes describing similarity between the sequences of the genes.

Though cross-mappability is a straightforward metric, its computation is non-trivial due to the size of the genome. We followed a systematic approach to compute genome-wide cross-mappabilities in practice. Following Derrien *et al.* ^4^, we define mappability of a *k*-mer as $\frac{1}{C_{k}}$, where *C _k_* is the number of positions where the *k*-mer maps to the genome with a tolerance of up to 2 mismatches. We computed exon- and UTR-mappability of a gene as the average mappability of all *k*-mers in exonic regions and untranslated regions, respectively. We used a collapsed gene model to generate *k*-mers where overlapped exons and overlapped UTRs were merged to form exonic and UTR regions, respectively. Then, mappability of a gene is computed as the weighted average of its exon- and UTR-mappability, weights being proportional to the total length of exonic regions and UTRs, respectively. Importantly, we only have to compute cross-mappability from genes with mappability < 1, as no *k*-mer from a gene with mappability = 1 will map to other regions of the genome (i.e. these will all result in cross-mappability of 0). Moreover, we need to consider only *k*-mers with mappability *<* 1 from a gene, as uniquely mapped *k*-mers will not map to other genes. So, we align all such *k*-mers from exonic and untranslated regions of each gene to the reference genome using Bowtie v1.2.2 ^18^, tolerating up to 2 mismatches, and then count the number of *k*-mers whose alignment start within exonic or untranslated regions of every other gene to compute cross-mappability with each gene genome-wide ( Figure 1B).

The length *k* may be tuned to match particular read length or alignment method. Here, if the value of *k* is not mentioned for *k*-mers, the default value of *k* is 75 for exons and 36 for UTRs. We used a smaller *k* for UTRs than for exons because UTRs are generally shorter than exons. Mappability of a gene and cross-mappability to/from a gene is undetermined if all the exons of the gene are shorter than 75 bp and all the UTRs are shorter than 36 bp.

We computed genome-wide mappability and cross-mappability for human genome hg19 using annotations from Gencode v19 ^19^. 26,200 (out of 57,820) genes had at least one *k*-mer cross-mapping to/from another gene. There were 31,167,448 gene pairs (0.93%) that were cross-mappable (cross-mappability > 0). Supplementary Figure 1A shows the cross-mappability distribution. We found that 2.45–4.92% of gene pairs expressed and quantified in five tissues of the GTEx v7 data were cross-mappable ( Supplementary Figure 1B). We also computed the same set of resources for human genome GRCh38 using annotations from Gencode v26, all of which are publicly available ^17^.

### Data

We downloaded fully processed, filtered and normalized gene expression data used in GTEx eQTL analysis from the GTEx portal ( www.gtexportal.org). For this study, we focused on gene expression data from 5 tissues: whole blood, skeletal muscle, thyroid, sun-exposed skin, and testis. We also obtained covariates including 3 genotype PCs representing ancestry, sex, genotyping platform, and PEER factors ^20^ as released in GTEx v7. GTEx aligned 76-bp paired-end reads to the reference genome with STAR v2.4.2a ^21^, quantified gene expression levels with RNA-SeQC v1.1.8 ^22^ using uniquely mapped reads aligned in proper pairs and fully contained within exon boundaries where each alignment must not contain more than six non-reference bases. We downloaded genotype data from GTEx release v7 from dbGaP (accession number: phs000424.v7.p2).

We also collected genotype, processed RNA-seq, and covariate data for the DGN cohort, which is available through the National Institute of Mental Health (NIMH) Center for Collaborative Genomic Studies on Mental Disorders. DGN aligned the reads to the reference genome using TopHat ^23^ and quantified gene expression levels using HTSeq ^24^. Latent factors inferred from the expression data have already been regressed out of the processed DGN data to address hidden confounders, as described in 13. Gene symbols were mapped to Ensembl gene ids using Gencode v19.

We downloaded the list of trans-eQTLs in 33 cancer types detected by PancanQTL ^25^ from http://bioinfo.life.hust.edu.cn/PancanQTL. For consistency with our study, we used trans-eQTLs where the variant and the gene were on different chromosomes, and the gene symbols were mapped to unique Ensembl gene ids according to Gencode v19.

### Trans-eQTL detection

For trans-eQTL analysis, we selected autosomal variants with MAF ≥ 0.05 that did not fall in a repeat region as annotated by the UCSC RepeatMasker track ^26^. We tested trans-eQTL association for each inter-chromosomal variant-gene pair using Matrix-eQTL’s linear model test ^27^. For GTEx, three genotype PCs, genotyping platform, sex, and PEER covariates estimated by GTEx were used as covariates in Matrix-eQTL. We computed the false discovery rate using the Benjamini-Hochberg method within each tissue. The covariates used for trans-eQTL replication in DGN were three genotype PCs, sex and age, as the expression data already had latent factors regressed out.

### Co-expression analysis

We quantified co-expression of a pair of genes as the absolute Pearson correlation ( *|r|*) between expression levels of the genes across all available samples. For GTEx, we regressed out all covariates including PEER factors before co-expression analysis. For DGN, we used the corrected data which also regresses out latent factors.

## Results

### Effect of cross-mappability on trans-eQTL detection

To investigate the effects of alignment errors on trans-eQTL detection, we performed a standard trans-eQTL analysis using data from the GTEx project for five human tissues. For this study, we categorized an eQTL as “cis” if the variant is within 1Mb of the transcription start site (TSS) of the gene, and “trans” if they are on different chromosomes, approximating the regions where cis and trans mechanisms are likely to occur. We call a trans-eQTL “cross-mappable” if any gene within 1Mb of the identified trans-eQTL variant cross-maps to the trans-eQTL target gene. The cross-mappable trans-eQTLs represent suspicious hits that could potentially arise simply due to alignment errors, although cross-mappability does not definitively establish that any individual trans-eQTL is a false positive.

**Figure 2.**
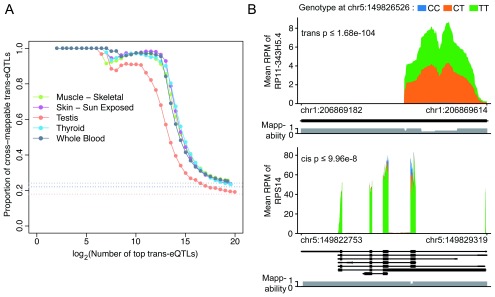
Effect of cross-mappability on trans-eQTLs in GTEx. **A**) Fraction of cross-mappable trans-eQTLs among the top significant variant-gene pairs (ordered by increasing FDR) in each tissue (color). Each dotted horizontal line represents the background cross-mappable rate in a given tissue. **B**) An example of likely false positive trans association between the variant chr5:149826526 and the gene RP11- 343H5.4. The coverages (reads per million, RPM) of the trans-eGene RP11-343H5.4 (top) and its cross-mapping gene RPS14 (bottom) in Thyroid are shown along with their exons and UTRs (black lines below the coverage plot), and mappability of 75-mers. The regions of mappability less than 1.0 have sequence similar between the two genes.

We identified 19,348 unique trans-eQTLs (variant-gene pairs) at FDR *≤* 0.05 from five tissues corresponding to 14,785 unique SNPs and 1,419 unique genes. Notably, a large majority (75.14%) of these statistically significant trans-eQTLs were cross-mappable. Furthermore, the cross-mappable eQTLs tended to be the most highly significant (ordered by increasing p-value, Figure 2A). In GTEx tissues, 90.8–97.3% of top 1000 trans-eQTLs were cross-mappable, compared to a background rate of 19.1–25.6% (based on all tested variant-gene pairs). The fraction of cross-mappable trans-eQTLs is very high even when we restrict our analysis to protein-coding genes or to genes with mappability ≥ 0.8 ( Supplementary Figure 2A–C).

We observed a similar pattern in the trans-eQTLs reported from RNA-seq data of 33 cancer types ^25^ ( Supplementary Figure 2D). We also observed that randomly selected variant-gene pairs susceptible to cross-mapping yield more trans-eQTLs than randomly selected pairs with no cross-mapping potential ( Supplementary Figure 3). Overall, the high fraction of cross-mappable eQTLs among the top associations in multiple tissues and multiple datasets indicates that alignment errors could be a major source of artifacts, dominating legitimate trans-eQTLs. It is also important to note that filtering such prevalent potential false-positives necessitates re-assessing FDR. For example, while 4,809 trans-eQTLs with no evidence of cross-mapping (corresponding to 969 unique genes) were among the 19,348 hits from the original scan of GTEx, only 2,456 (corresponding to 228 unique genes) would appear significant if FDR were reassessed after filtering cross-mapping hits.

When we further analyzed the composition of the 19,348 significant naive trans-eQTLs, we observed a majority (>70%) of cross-mappable eQTLs corresponded to pseudogene targets. The non-cross-mappable eQTLs contained far fewer pseudogene targets (30%, Supplementary Figure 4). Likewise, we observed that more than 85% of eQTLs corresponding to pseudogenes were cross-mappable. Due to sequence similarity between pseudogenes and their corresponding parent genes, this is not surprising and could be due to alignment errors. One simple preventative measure in trans-eQTL studies would be to simply exclude pseudogenes entirely. However, 42.4% of eQTLs corresponding to protein-coding genes were also cross-mappable, which still exceeded expectation, and the top hits remained enriched for cross-mapping errors as noted above.

We investigated one GTEx trans-eQTL in greater detail for illustration – variant: chr5:149826526 and gene: RP11-343H5.4 (ENSG00000224114) – which was significant in each of 5 GTEx tissues. RP11-343H5.4 is a pseudogene on chromosome 1. In the coverage plots of the gene, we noticed that reads were aligned to only a fraction of the exonic region of the gene; if the gene were truly expressed, we would expect reads being mapped across the whole exon ( Figure 2B). RP11-343H5.4 is cross-mappable with RPS14 (ENSG00000164587), a protein-coding gene in chromosome 5 near the putative trans-eQTL variant. There was also a cis-association between the variant and RPS14. *k*-mers from RPS14 indeed map to the region within RP11-343H5.4, where we observed a non-zero number of reads. Interestingly, in this case, read mapping appears to be genotype-dependent - the variant at chr5:149826526 alters sequence such that it would lead to reads from RPS14 uniquely, but likely incorrectly, mapping to RP11-343H5.4.

**Figure 3.**
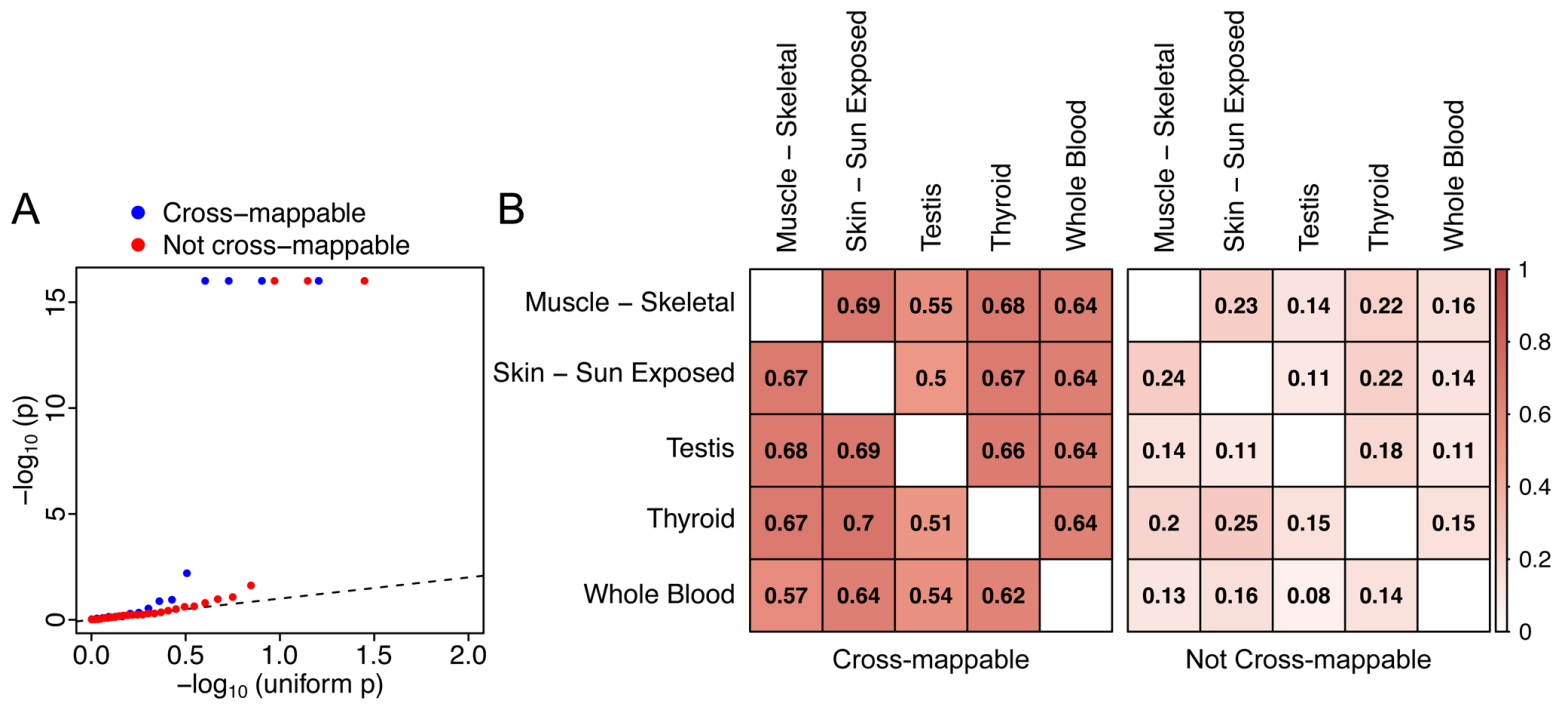
Trans-eQTL replication. ( **A**) Q-Q plot, replication p-values from DGN for variant-gene pairs discovered in GTEx Whole Blood, grouped by cross-mappability. ( **B**) The fraction of significant eQTLs in each GTEx tissue (row) replicated in another tissue (column) at FDR ≤ 0.05, for cross-mappable eQTLs (left) and not cross-mappable eQTLs (right).

Finally, we found that cross-mappable eQTLs, which we believe to be enriched for false-positives, are highly replicable between datasets. This misleading replication occurs because it is driven by the underlying sequence of the genome, and similar alignment errors frequently occur regardless of tissue and study. We showed this by measuring the replication between the significant trans-eQTLs detected at FDR ≤ 0.05 from whole blood from GTEx and whole blood data from the DGN study ^13^. To avoid the effects of linkage disequilibrium, we tested for trans-association in DGN only for the best variant per GTEx trans-eQTL gene (with the lowest p-value in GTEx), where both the variant and the gene were present in the DGN data. At FDR ≤ 0.05, only 10.71% (3 out of 28) non-cross-mappable trans-eQTLs were replicated in DGN while 31.25% (5 out of 16) cross-mappable trans-eQTLs were replicated. The Q-Q plot in Figure 3A shows that cross-mappable trans-eQTLs were more likely to be replicated compared to non-cross-mappable ones. We observed the same phenomenon when we attempted to replicate significant trans-eQTLs detected from one GTEx tissue in other GTEx tissues. On average, 63.0% (range: 50.3–70.2%) and 16.3% (range: 7.6–25.1%) of cross-mappable and non-cross-mappable trans-eQTLs, respectively, were replicated ( Figure 3B). This suggests that replication of a trans-eQTL does not necessarily indicate a true positive. Overall, we suggest that regardless of replication, cross-mappable trans-eQTLs require further investigation to establish that they arise from biological regulation rather than alignment artifacts.

### Effect of cross-mappability in co-expression analysis

**Figure 4.**
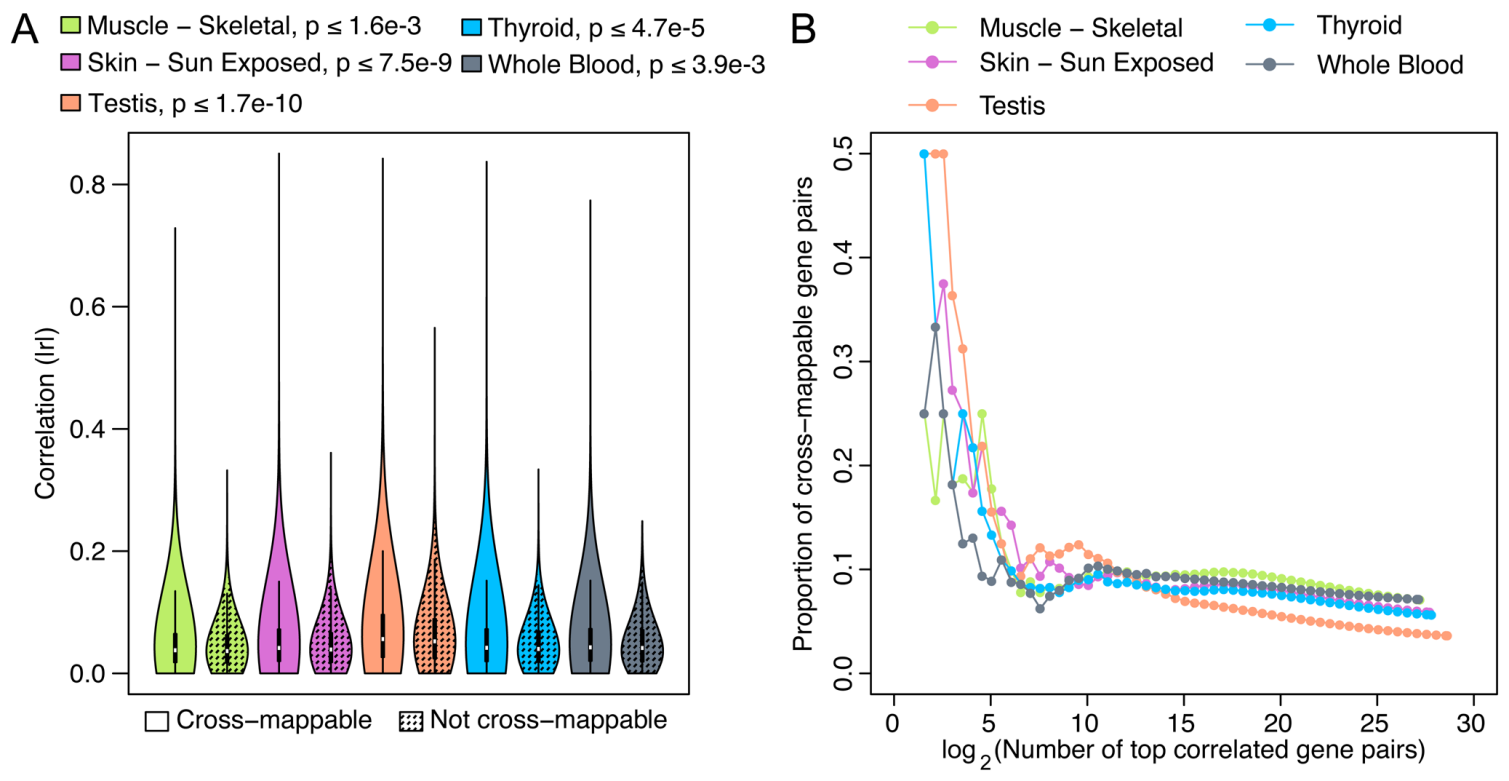
Effect of cross-mappability on co-expression. ( **A**) Comparison of co-expression between randomly drawn pairs of cross-mappable genes and not cross-mappable genes. Each violin plot shows the distribution of the absolute Pearson correlation (y-axis) between corrected gene expression levels of randomly drawn 10,000 gene pairs in a tissue (color). P-value of the Wilcoxon test to determine whether cross-mappable genes are more correlated than not cross-mappable genes in each tissue is shown in the legend. ( **B**) Fraction of top co-expressed genes that are cross-mappable and thus potential false positives.

Next, we evaluated evidence that alignment errors between genes can cause spurious correlation between gene expression levels (co-expression). If alignment errors did not affect co-expression analysis, we would expect that the distribution of pairwise correlation between cross-mappable genes would not deviate from that between non-cross-mappable genes. To test this, we used the gene expression data in five tissues from GTEx v7 after correction for covariates and latent confounders (see Methods). For each tissue, we selected a random set of 10,000 non-cross-mappable gene pairs and a random set of 10,000 cross-mappable gene pairs chosen with probability proportional to their cross-mappabilities (sampling probability proportional to cross-mappability ensures sampling from the whole cross-mappability range, as opposed to just from the massive number of low cross-mappability pairs). Then we computed the absolute Pearson correlation ( *|r|*) between expression levels of the genes in each randomly selected pair. We found that expression levels of cross-mappable genes were more correlated than expression levels of non-cross-mappable genes (median *p* across tissues ≤ 4.7 *×* 10 ^−5^, Wilcoxon rank-sum test, Figure 4A). The difference was more significant when uncorrected data were used (median *p ≤* 1.3 *×* 10 ^−74^, Supplementary Figure 5). We also observed that the correlation coefficient tends to increase with increasing levels of cross-mappability between genes ( Supplementary Figure 6), indicating a high rate of false co-expression in the most highly cross-mappable genes. The increased correlation between cross-mappable genes was observed even after discounting genes from same gene family ( Supplementary Figure 7), somewhat alleviating concerns that our observations were due to exclusively true functional relationships. We observed a similar pattern using data from an independent RNA-seq study, DGN ( Supplementary Figure 8).

To demonstrate the impact of this pattern on a realistic genome-wide co-expression analysis, we evaluated how many of the top-most correlated gene pairs in each GTEx tissue suffer from cross-mappability. We observed that cross-mappable pairs of genes are over-represented among the top hits, with gene pairs ordered by the absolute Pearson correlation after excluding pairs of genes whose genomic coordinates actually overlap ( Figure 4B, Supplementary Figure 9). Overall, the impact of cross-mappability on co-expression appears to be less than on trans-eQTL analysis, but the phenomenon may still require consideration when examining specific co-expressed gene pairs or enrichment patterns.

### Impact of alternative quantification and parameter settings

We have made several versions of our cross-mappability resources publicly available for the human genome (hg19 and GRCh38) ^17^, and also published code in Github ^16^. Researchers should carefully choose settings according to the study design and goals. Genome version and gene annotations can be directly matched, but other parameter choices such as *k* and the maximum number of mismatches allowed in alignment may affect the detection of false positives. Small values of *k* will produce more conservative cross-mappability scores, but large *k* may not correctly handle small exons or UTRs. For example, if 75-mers (instead of 36-mers) were used from UTRs, a smaller proportion of trans-eQTLs (67.2% instead of 75.14%) would appear as cross-mappable in GTEx, although cross-mappable trans-eQTLs would still tend to be most highly significant ( Supplementary Figure 10A). Similarly, increasing the number of mismatches allowed in *k*-mer alignment results in an increased number of cross-mappable trans-eQTLs ( Supplementary Figure 10B). For convenience, *k* and the number of mismatches are configurable in our software so that, if needed, one can compute cross-mappability scores with settings appropriate for a given study.

We also note that utilization of improved alignment and quantification methods to generate gene expression data may also be helpful to avoid false positives. For example, quantification of gene expression levels using RSEM ^28^, an expectation maximization based quantification tool, results in a smaller fraction of false positive trans-eQTLs (60.17%) than that using RNA-SeQC (75.14%). However, potential false positives due to cross-mappability still remain abundant in both trans-eQTL and co-expression studies ( Supplementary Figure 11).

## Discussion

Misalignment of short sequencing reads has the potential to induce false positives in association studies. For RNA-seq, both trans-eQTL and co-expression analyses are susceptible to these artifacts, related to false positives in microarray analysis due to probe cross-hybridization. This is readily apparent from the enrichment of processed pseudogenes among the top hits for such association studies, but misalignment can affect protein-coding genes as well. Our results demonstrate that trans-eQTL associations in a standard pipeline are dominated by potential false-positives due to sequence similarity and replication rates between studies may be artificially inflated due to this pattern. Additionally, genes with sequence similarity display more correlated expression levels, and mapping errors should be considered in co-expression analysis as well.

Our results do not imply that all instances of co-expression or trans-eQTL associations arising from genes with sequence similarity are in fact false positives. Genes with sequence similarity also sometimes have true functional relationships. Pseudogene transcripts may interact with coding transcripts, and some associations with pseudogene expression may reflect true regulatory relationships ^29^. Furthermore, the background (random) rate of sequence similarity between any two regions in the human genome is above zero; that is, a hit may occur between regions of sequence similarity by chance, even when no actual misalignment of reads has taken place. However, we believe the exceedingly high fraction of cross-mappable regions among trans-eQTLs from a naive analysis warrants suspicion that these hits are predominantly false positives. Researchers should consider their particular application and tolerance for false negatives and false positives when applying filters targeting alignment errors. Other information, such as base-level coverage plots and outside functional information can help disambiguate particular cases of interest.

Extensions, modifications, and other approaches related to this problem should also be considered. First, specifics of study design, and in particular sequencing read length, should be taken into account when using our data to filter potential false positives. If read length is much shorter or longer than our *k*-mer setting, our existing data may be insufficient and new mappability and cross-mappability estimates should be derived. In the initial resource provided, we used *k*-mer alignment to the genome, which does not directly handle splice junctions in transcriptomic data (and also limits appropriate *k*-mer length even for studies with longer reads). Alignment to the transcriptome or splice-aware alignment may offer future improvements, but computational cost and inaccuracies due to incorrect annotation will have to be evaluated. Our observations and methods may be relevant to analyses of other functional genomic data as well, including detection of interactions from HI-C, and detection of associations with data types such as ATAC-seq or ChIP-seq. Other approaches, such as filtering reads themselves before quantification can also be applied if raw reads rather than quantified data are available and tractable ^12^.

Our evaluation provides evidence that misalignment of reads should be considered as a potential source of false positives in association studies, particularly for trans-eQTL analysis. The resources we provide can be used directly to filter potential false positives, or the ideas presented may be tuned and adapted to new studies and data types.

## Data availability

### Underlying data

Pre-computed cross-mappability resources for human genomes (hg19 and GRCh38) are available on figshare, DOI: 10.6084/m9.figshare.c.4297352.v4 ^17^. GTEx (v7) expression and covariate data are available from www.gtexportal.org. GTEx (v7) genotype data are available from dbGap (accession number: phs000424.v7.p2). DGN data are available by application through NIMH. Other data, including annotations and intermediate results, required to reproduce analyses in the manuscript are available on figshare, DOI: 10.6084/m9.figshare.7309625.v2 ^30^.

### Extended data

Supplementary Figure 1– Supplementary Figure 11 are available on figshare, DOI: 10.6084/m9.figshare.7359539.v2 ^31^.

**Supplementary Figure 1. Cross-mappability statistics.** (A) Distribution of cross-mappability between cross-mappable pairs of genes, restricted to gene pairs with cross-mappability > 0, using Gencode v19 annotations on human genome hg19. (B) Background percentage of cross-mappable gene pairs between all available expressed genes in GTEx data, categorized by tissue. For both panels, directed gene pairs were used; i.e., (Gene A, Gene B) and (Gene B, Gene A) pairs were considered different.

**Supplementary Figure 2. Cross-mappability among top trans-eQTLs.** Detected (A) using protein-coding genes in GTEx, (B) using genes with mappability ≥ 0.8 in GTEx, (C) using protein-coding genes with mappability ≥ 0.8 in GTEx, and D) by PancanQTL where unique eQTLs were ordered by lowest p-value across all cancer types.

**Supplementary Figure 3. Large number of trans-eQTLs among random cross-mappable gene pairs.** We tested for trans-eQTLs taking the same number of random variant-gene pairs in 3 different categories: 1) Not cross-mappable, 2) Cross-mappable, and 3) Cross-mappable (Top). In the first category, we randomly selected 1,000 not cross-mappable gene pairs (g1, g2) where g1 and g2 were on different chromosome and there was at least one variant near g1 (within 1Mb of the TSS of g1), then selected the best cis-variant s (with lowest p-value) for g1, and finally tested for trans-association between s and g2. Variant-gene pairs for other two categories were selected in a similar way as the first category except that the gene pairs were cross-mappable (crossmap(g1, g2) > 0) in the second category, and highly cross-mappable (among top 10,000 cross-mappable pairs) in the third category. The above plot shows the number of significant trans-eQTLs (y-axis) detected at a given FDR (x-axis) in each category (line marker) in each tissue (color).

**Supplementary Figure 4. Composition of trans-eQTLs.** (A) Representation of gene types among trans-eQTL target genes, categorized by cross-mappability. (B) Proportion of cross-mappable eQTLs categorized by gene type. Only the four most frequent gene types in trans-eQTL hits are shown. (C) Among trans-eQTLs with a pseudogene target gene, quantification of different pseudogene sub-types, categorized by cross-mappability. Pseudogene sub-types were identified from the Gencode v26 annotation, as sub-types are not available in Gencode v19. The five most frequent types among trans-eQTL hits are shown.

**Supplementary Figure 5. Correlation between random gene pairs using uncorrected data.** Each violin plot shows the distribution of absolute Pearson correlation (y-axis) between uncorrected gene expression levels of 10,000 randomly drawn gene pairs in a tissue (color). The p-value of the Wilcoxon test to determine whether cross-mappable genes are more correlated than not cross-mappable genes in each tissue is shown in the legend.

**Supplementary Figure 6. Correlation between random gene pairs increases with cross-mappability.** Gene pairs available in each tissue were categorized into 22 groups (x-axis) based on quantiles. A quantile group " *q*_1_ − *q*_2_( *n*)" represents gene pairs of ( *q*_1_ * 100, *q*_2_ * 100]-th percentile of cross-mappability with a total of *n* pairs. In order to visualize the impact of the highest range of cross-mappability, the rightmost nine quantile groups were selected in such a way that each contains about a certain number of pairs: (from right) 2,000, 2,000, 2,000, 2,000, 2,000, 5,000, 10,000, 25,000, 50,000. The leftmost quantile group "0" represents gene pairs which are not cross-mappable. From each group, 1,000 gene pairs were randomly selected where the probability of drawing a pair was proportional to its cross-mappability. Each violin plot shows the distribution of absolute Pearson correlation (y-axis) between corrected expressions of the genes in each pair.

**Supplementary Figure 7. Increased correlation between cross-mappable genes is not exclusively due to sequence similarity between genes from same gene family.** Here, two genes in the same HGNC gene family were artificially excluded from cross-mappable pairs. We computed the absolute Pearson correlation between gene pairs within different groups as described in Figure 4A and Supplementary Figure 6. Note: gene family information was downloaded from www.genenames.org. (A–B) Comparison of co-expression between 10,000 randomly drawn pairs of cross-mappable and not cross-mappable genes in Muscle – Skeletal (A) and Whole Blood (B). (C–D) Random correlation between genes in Muscle – Skeletal (C) and Whole Blood (D).

**Supplementary Figure 8. Co-expression analysis using gene expression data from DGN.** (A) Comparison of co-expression between 10,000 randomly drawn cross-mappable and non-cross-mappable gene pairs. (B) Random correlation between genes in DGN increases with cross-mappability.

**Supplementary Figure 9. Fraction of gene pairs with cross-mappability ≥ 100 among the top co-expressed genes, categorized by GTEx tissues.**

**Supplementary Figure 10. Effects of varying k-mer length and the number of mismatches allowed.** Cross-mappability among the top GTEx trans-eQTLs when (A) 75-mers (instead of 36-mers) from UTRs were used, (B) a maximum of 3 (instead of 2) mismatches were allowed. 67.2% and 76.1% of the significant trans-eQTLs remain cross-mappable in (A) and (B), respectively, compared to 75.14% using 75-mers from exons and 36-mers from UTRs with 2 mismatches in the original analysis. In both cases, cross-mappable trans-eQTLs still tend to be the most highly significant.

**Supplementary Figure 11. Effects of EM-based quantification methods.** We downloaded transcript-level quantifications based on RSEM ^28^ from GTEx and derived gene-level TPMs using the tximport package ^32^ in R. We used same set of genes and samples as available in the regular gene-level quantifications used in our main GTEx eQTL anaylysis. Following the GTEx pipeline, we normalized gene expression values between samples using TMM as implemented in edgeR ^33^ and then performed an inverse normal transformation of expression values for each gene across all samples. (A) We computed the number of cis-eGenes on two chromosomes (chr7 and chr14) for different numbers of PEER factors, estimated from RSEM-quantifed gene-level data for each tissue. As in the standard GTEx Consortium analysis, the number of cis-eGenes tended to increase with the number of PEER factors. With no clear difference in behavior, we opted to use the same number of PEER factors as used in the standard analysis, along with three genotype PCs, genotyping platform and sex. (B) The plot shows trans-eQTL p-values using RSEM-quantified data (y-axis) vs. standard RNA-SeQC GTEx data (x-axis), for each significant trans-eQTL (point) in whole blood. Here, the color represents whether the eQTL is cross-mappable or not, and the symbol represents whether the target gene is a pseudogene or not. The majority of points lie close to the diagonal line, indicating the two quantification methods give mostly similar results, regardless of gene type and including the majority of cross-mappable hits. A few points along the horizontal line at y=0 shows that a small fraction SNP-gene pairs are no longer significant with RSEM-quantified gene expression and most of them are cross-mappable pseudogenes. Thus RSEM offers some modest improvement, but does not resolve the majority of problematic hits. (C) We computed trans-eQTLs genome-wide using RSEM-quantified data. A total of 27,035 trans-eQTLs were detected at FDR ≤ 0.05, 60.17% of which were cross-mappable compared to 75.14% with RNA-SeQC-quantified data. The plot shows the fraction of cross-mappable trans-eQTLs among the top significant variant-gene pairs (ordered by increasing FDR) in each tissue (color). Again, we observe a modest improvement from RSEM. (D) Fraction of top co-expressed genes that are cross-mappable and thus potential false positives. Cross-mappable gene pairs still appear abundant in most correlated genes of multiple tissues.

## Software availability

**GitHub repository to compute cross-mappability:** https://github.com/battle-lab/crossmap.

**Archived code at time of publication:** https://doi.org/10.5281/zenodo.2602096 ^16^.

**License:** GPL-3.

**GitHub repository to replicate analyses in the manuscript:** https://github.com/battle-lab/crossmap_analysis.

**Archived code at time of publication:** https://doi.org/10.5281/zenodo.2602170 ^34^.

**License:** GPL-3.
