# Supplementary material for: False positives in trans-eQTL and co-expression analyses arising from RNA-sequencing alignment errors
Source: F1000Res. 2019 Apr 8;7:1860. Originally published 2018 Nov 28. [Version 2] doi: 10.12688/f1000research.17145.2 (PMC6305209; doi:10.12688/f1000research.17145.2)
Supplement: Peer review history

# Peer review: Reviewer response for version 2

**DOI:** 10.5256/f1000research.20603.r46860

**Published:** 2019-07-15

**Authors:** Michael I. Love

**Affiliations:** 1 Department of Biostatistics, The University of North Carolina at Chapel Hill, Chapel Hill, NC, USA

In their revision, the authors addressed a comment in my initial report that EM-based quantification methods may, to some degree, alleviate the cross-mapping problem. In their Supplementary Figure 11, they show that using RSEM to estimate expression removes some of the false positive trans eQTL (points on the x-axis), but that the majority of FP trans eQTL persist (points on the diagonal). They have therefore addressed all of my initial comments/concerns. The added distinction between mappability and cross-mappability is also useful.

I confirm that I have read this submission and believe that I have an appropriate level of expertise to confirm that it is of an acceptable scientific standard.

# Peer review: Reviewer response for version 1

**DOI:** 10.5256/f1000research.18744.r41238

**Published:** 2018-12-27

**Authors:** Aaron R. Quinlan

**Affiliations:** 1 Department of Human Genetics, USTAR (Utah Science Technology and Research) Center for Genetic Discovery, University of Utah, Salt Lake City, UT, USA

Most assays that leverage high-throughput DNA sequencing ultimately involve counting molecules that align to genome loci. The observed counts are, in turn, used as proxies for some underlying cellular phenomenon. If the process of sequence alignment is fundamentally biased, so too will be the observations and potential conclusions drawn. 

 In this manuscript, Saha and Battle explore the important role of sequence similarity between genes ("cross-mapping") on the spurious detection of trans eQTLs and gene co-expression. In particular, they argue that up to 75% of trans e-QTLs detected with standard methods could be spurious owing to systematic mapping (and thus both downstream read-counting and subsequent expression measures) artifacts caused by sequence similarity. This manuscript is well-structured, easy to read, and provides yet another warning that properly accounting for mapping and alignment artifacts is critical genomic research. The approach and analyses are sound overall. However, there are a few area that would benefit from clarification, and some technical aspects that warrant greater detail and perhaps further analysis.

1. Choice of k-mer size. While the choice of k=75 for exons and k=36 for UTRs is certainly reasonable, one wonders whether these choices provide the greatest power to detect and quantify cross-mappability. Were analyses conducted to choose these k-mer sizes empirically? It would also be nice to add methods describing from which gene models the k-mers were derived. Furthermore, 5' UTRs are, on average, only slightly smaller than the typical exon, and 3' UTRs are actually larger on average (https://gist.github.com/arq5x/0d44bae195fc6260984ee01fd253712c). Therefore, it is not clear that k=36 for UTRs versus k=75 for exons is well-justified.
2. When reading the manuscript, one naturally wonders whether the effects revealed by cross-mappability could be detected via existing "mappability" scores from ENCODE and others. It would helpful to have an explicit discussion and/or analysis of how this metric differs.
3. By my reading and interpretation of Figure 1B, k-mers spanning exon/exon junctions were not modeled. This is perhaps critical given the results describing an enrichment of cross-mappable eQTLs corresponding to processed pseudogenes.
4. Echoing the point raised by Rob Patro, it is important to understand how the gene expression qualification was done for GTeX and discuss how these methods impact cross-mappability analysis.  
5. Mappability is defined as 1/C_k where C_k is the number of genomic loci to which the k-mer aligns with <= 2 mismatches.  Given sequencing errors, polymorphism, and the fact that RNA-seq is actually cDNA-seq, one might be concerned that allowing more mismatches (and/or INDEL errors) would more properly model cross-mappability. While perhaps a minor concern, it would be nice to know how much the scores and resulting cross-mappability table differ with tolerance for larger edit distances.
6. In the results describing an enrichment of spurious trans eQTLs associated with cross-mapping pseudogenes, it is unclear whether the authors are referring to all types of pseudogenes or solely processed pseudogenes.  Crossmapping could arise from both "classic" (i.e., duplicated owing to NAHR) and "processed" (i.e., mature RNAs duplicated/inserted into the genome). However, the effect of excluding k-mers that span exon/exon boundaries from the cross-mappability would have a differential effect on these two types of pseudogenes. It would be helpful to be explicit in which types of pseudogenes are being discussed.
7. SNPs were excluded from evaluation if they overlapped annotations in the RepeatMasker track.  I would argue that SNPs should (and typically would be) excluded if they lie within segmental duplications. Such regions also harbor genes with a high degree of paralogy (because of the ancestral duplication) and if excluded, may have a substantial impact on the results. For example, I suspect many "classic" pseudogenes would also be excluded were SNPs in segmental duplications excluded. While I have never conducted a trans eQTL analysis, I suspect that removal of segdups is standard practice owing to the inherently high paralogy between duplicated regions.

I confirm that I have read this submission and believe that I have an appropriate level of expertise to confirm that it is of an acceptable scientific standard.

# Response

**Published:** 2019-04-09

**Authors:** Ashis Saha

**Affiliations:** Johns Hopkins University, USA

***Thank you for reviewing our paper.  Please see our responses inline in bold and italic font following your comments in standard font.***

 Most assays that leverage high-throughput DNA sequencing ultimately involve counting molecules that align to genome loci. The observed counts are, in turn, used as proxies for some underlying cellular phenomenon. If the process of sequence alignment is fundamentally biased, so too will be the observations and potential conclusions drawn. 

 In this manuscript, Saha and Battle explore the important role of sequence similarity between genes ("cross-mapping") on the spurious detection of trans eQTLs and gene co-expression. In particular, they argue that up to 75% of trans e-QTLs detected with standard methods could be spurious owing to systematic mapping (and thus both downstream read-counting and subsequent expression measures) artifacts caused by sequence similarity. This manuscript is well-structured, easy to read, and provides yet another warning that properly accounting for mapping and alignment artifacts is critical genomic research. The approach and analyses are sound overall. However, there are a few area that would benefit from clarification, and some technical aspects that warrant greater detail and perhaps further analysis.

***>>  Thank you for your positive assessment of our approach.***

 1. Choice of k-mer size. While the choice of k=75 for exons and k=36 for UTRs is certainly reasonable, one wonders whether these choices provide the greatest power to detect and quantify cross-mappability. Were analyses conducted to choose these k-mer sizes empirically? It would also be nice to add methods describing from which gene models the k-mers were derived. Furthermore, 5' UTRs are, on average, only slightly smaller than the typical exon, and 3' UTRs are actually larger on average (https://gist.github.com/arq5x/0d44bae195fc6260984ee01fd253712c). Therefore, it is not clear that k=36 for UTRs versus k=75 for exons is well-justified.

***>>  We appreciate this consideration. To alleviate such concerns regarding the value of k, we have provided options to select appropriate k for exons or UTRs in our code for users that would prefer a different setting. Our choice of k=75 was originally guided by the RNA-seq read-length of the GTEx data, and a reduced length for UTRs based on manual investigation of our top hits suggesting that k=75 for UTRs left too many questionable instances unfiltered.  However,  because there is no gold standard for which trans-eQTLs are truly false positives, we did not think of a way to tune this more closely.***

***To provide further insight, we have also now computed cross-mappability using 75-mers from both exons and UTRs and shared these resources publicly. We also shared resources with 50-mers from exons and 36-mers from UTRs.  Using 75-mers from both exons and UTRs, 67.2% of unique trans-eQTLs appeared as cross-mappable, compared to 75.1% when 75-mers from exons and 36-mers from UTRs were used. We believe it is likely missing some false-positives at this larger k, but we cannot prove this for certain.  Cross-mappable eQTLs still tend to be the most highly significant. We have added discussion of the issue in the manuscript along with Supplementary Figure 10A. ***

- ***“Researchers should carefully choose settings according to the study design and goals. Genome version and gene annotations can be directly matched, but other parameter choices such as k and the maximum number of mismatches allowed in alignment may affect the detection of false positives. Small values of k will produce more conservative cross-mappability scores, but large k may not correctly handle small exons or UTRs. For example, if 75-mers (instead of 36-mers) were used from UTRs, a smaller proportion of trans-eQTLs (67.2% instead of 75.14%) would appear as cross-mappable in GTEx, although cross-mappable trans-eQTLs would still tend to be most highly significant (Supplementary Figure 10A)”***

***We also clarified that we used a collapsed gene model to generate k-mers where overlapped exons and overlapped UTRs were merged to form exonic and UTR regions, respectively.  A locus that overlaps both an annotated UTR and an annotated exon will be considered as both.***

 2. When reading the manuscript, one naturally wonders whether the effects revealed by cross-mappability could be detected via existing "mappability" scores from ENCODE and others. It would helpful to have an explicit discussion and/or analysis of how this metric differs.

***>>  Thank you for raising the point. Existing "mappability" scores (described by either Derrien et al. or Karimzadeh et al.) correspond to a single genomic region (e.g., gene). In contrast, our "cross-mappability" score corresponds to a pair of genes. While mappability score tells about how unique the sequences of a gene (or a region) is, it does not talk about how similar the sequences of a given pair of genes are. A gene with mappability=1 (mappability = 1 for every k-mer of the gene) will have cross-mappability = 0 from/to any other gene (we directly used this property to optimize our computation of cross-mappability genome-wide).  However, cross-mappability between two genes with mappability < 1 cannot be revealed by mappability. We now described the difference between these two metrics in the revised manuscript.  Genes with mappability < 1 may indeed cross-map with other genes, or may have sequence similarity with non-transcribed regions of the genome, in which case they would not appear cross-mappable in our analysis.***

- ***"Notably, existing mappability scores [4, 5] correspond to a single region (or gene) describing uniqueness of the sequences of the region in the genome, our cross-mappability score corresponds to a pair of genes describing similarity between the sequences of the genes."***

 

 3. By my reading and interpretation of Figure 1B, k-mers spanning exon/exon junctions were not modeled. This is perhaps critical given the results describing an enrichment of cross-mappable eQTLs corresponding to processed pseudogenes.

***>>  We appreciate your concern. We do think accounting for k-mers spanning exon-exon junctions would offer improvements of cross-mappability, and we plan to investigate this further. Please see our response to Rob Patro for details.***

 4. Echoing the point raised by Rob Patro, it is important to understand how the gene expression qualification was done for GTeX and discuss how these methods impact cross-mappability analysis.  

***>>  We agree. We performed additional analyses using RSEM-quantified gene expressions and we still observed potential false positives in our analyses. Please see our response to Mike Love for details along with new Supplementary Figure 11.***

 5. Mappability is defined as 1/C_k where C_k is the number of genomic loci to which the k-mer aligns with <= 2 mismatches.  Given sequencing errors, polymorphism, and the fact that RNA-seq is actually cDNA-seq, one might be concerned that allowing more mismatches (and/or INDEL errors) would more properly model cross-mappability. While perhaps a minor concern, it would be nice to know how much the scores and resulting cross-mappability table differ with tolerance for larger edit distances.

***>>  This is a good point. The number of mismatches is a configurable parameter of our code, and one can compute cross-mappabilities with any desired value. The cross-mappability scores may increase if the allowed number of mismatches is increased. We have now also added an additional analysis with <=3 mismatches (Bowtie v1 allows max 3 mismatches) along with new Supplementary Figure 10B. Maximum 3 mismatches results in a slightly higher percentage of cross-mappable trans-eQTLs than maximum 2 mismatches (76.1% vs. 75.14%).***

- ***“Similarly, increasing the number of mismatches allowed in k-mer alignment results in an increased number of cross-mappable trans-eQTLs (Supplementary Figure 10B)”***

6. In the results describing an enrichment of spurious trans eQTLs associated with cross-mapping pseudogenes, it is unclear whether the authors are referring to all types of pseudogenes or solely processed pseudogenes.  Crossmapping could arise from both "classic" (i.e., duplicated owing to NAHR) and "processed" (i.e., mature RNAs duplicated/inserted into the genome). However, the effect of excluding k-mers that span exon/exon boundaries from the cross-mappability would have a differential effect on these two types of pseudogenes. It would be helpful to be explicit in which types of pseudogenes are being discussed.

***>>  This is a good suggestion. It would be nice to see how different types of pseudogenes contribute to cross-mappability. Gencode v19 annotation does not distinguish among different types of pseudogenes, so we mapped the types of pseudogenes from Gencode v26 annotation, which should be sufficiently accurate for aggregate characterization. Our results show that processed pseudogenes are most common source of cross-mappability, while other pseudogenes do also contribute to cross-mappability. We added an extra plot in Supplementary Figure 4 showing the representation of different types of pseudogenes in trans-eQTLs with pseudogene targets.***

 7. SNPs were excluded from evaluation if they overlapped annotations in the RepeatMasker track.  I would argue that SNPs should (and typically would be) excluded if they lie within segmental duplications. Such regions also harbor genes with a high degree of paralogy (because of the ancestral duplication) and if excluded, may have a substantial impact on the results. For example, I suspect many "classic" pseudogenes would also be excluded were SNPs in segmental duplications excluded. While I have never conducted a trans eQTL analysis, I suspect that removal of segdups is standard practice owing to the inherently high paralogy between duplicated regions.

***>> This is a good point. We do mask SNPs based on RepeatMasker, but this only covers a subset of segdups.  In general for our false positives, the SNP is actually not within a segdup itself (segdup SNP eQTLs display a slightly higher rate of cross-mappability than background - 83% vs 75%) . The classic pattern is a SNP in the cis-region of one gene appearing associated with a distant region similar to the cis-gene (potentially a segdup in some cases), but the region of similarity does not include the SNP locus.  Excluding pseudogenes and/or segdups from candidate genes in a trans-eQTL study is certainly one option.  Making SNPs or excluding genes in segdups does not appear to be standard practice to the best of our knowledge, nor does it cover every likely false positive we observe, but is a change worth considering to standard trans-eQTL pipelines.***

# Peer review: Reviewer response for version 1

**DOI:** 10.5256/f1000research.18744.r41237

**Published:** 2018-12-21

**Authors:** Rob Patro

**Affiliations:** 1 Department of Computer Science, Stony Brook University, Stony Brook, NY, USA

In this manuscript, Saha and Battle describe how errors in the genomic alignment of RNA-seq data can confound specific downstream analyses. Specifically, the focus on the discovery of trans-eQTLs and on co-expression analysis. Surprisingly, they find that, when a "naive" pipeline is used for trans-eQTL discovery, up to 75% of the trans-eQTL events detected may be false positives resulting from cross-mappability (the type of alignment error they discuss and characterize). In addition to describing this phenomenon, and demonstrating its effect on trans-eQTL and co-expression analysis, the authors also propose a new "cross-mappability" score, which allows one to map out which genes in a reference are likely to suffer from the types of spurious alignments, and subsequently, spurious correlations, that are described. The idea of cross-mappability seems a useful and logical extension of the mappability concept, where one is instead interested in which pairs or groups of genes share homologous sequence. The authors also provide pre-computed cross-mappability scores for hg19 and GRCh38.

 The paper is well-written, and the issues that the authors raise are important ones. It suggests that researchers should be cautious in interpreting the results of eQTL and co-expression analyses, and, importantly, provides them with tools to reassess their data and help control for the strong potential confounding factor of cross-mappability. I believe this is an important contribution.

 My main questions and comments about the manuscript concern cross-mappability scores, and the alignment errors that lead to the observed problems.

  

- Though the authors only explore the effect of alignment errors on eQTL and co-expression analysis, it seems that these types of issues could affect most analyses involving spliced-mapping of RNA-seq data to the reference genome. Specifically, the type of alignment errors illustrated in Figure 1 (A) would affect even basic expression quantification, let-alone co-expression analysis. This is particularly true for reads where this effect persists even when one considers only reads aligned uniquely by the tool. What would cause the aligner to return only a single (incorrect) locus for the read when multiple equally-good alignments should exist? Are the alignments contiguous at one locus but spliced at the other, or is the manner in which the read would align to the "true" and "spurious" locus different? Interestingly, it seems as though the cross-mappability map could act as a sort of homology table 1 that might even be useful for correcting these spurious alignments, or at least suggesting the true locus as an equally-good match.
- Given that cross mappability is computed by mapping specific k-mers back to the genome (allowing up to 2 mismatches) using Bowtie, how does it deal with accounting for k-mers that span splicing junctions?  It seems to me that the specific case where reads map to pseudogenes rather than what is presumed to be the true (protein-coding) locus of the read could be explained by regions of the genome that are contiguous (un-spliced) in the pseudogene, but which span a splicing junction in the protein coding gene. If the cross-mappability score doesn't account for the cross-mappability of k-mer that may span splice junctions, then it seems it might miss such important cases. However, given that the score is computed assuming some known annotation, it would be possible to explicitly extract appropriately-sized contexts around each known splicing junction, and to include them into the reference that Bowtie maps against when computing the cross-mappability scores. How would the cross-mappability scores change if they also accounted for junction-spanning k-mers rather than just genomically contiguous k-mers? 
- Related to the above point, but thinking in the other direction, might the cross-mappability scores be too "conservative" in some cases? Specifically, the scores are computed using k-mers that are the length of relatively short reads for exons, and k-mers that are much shorter than typical reads for UTRs. However, the ambiguity of these sequences individually may not be sufficient to induce a false alignment when the reads being processed are paired-end reads.  In that case, one would expect misalignments to require that there be sequence ambiguity supporting both ends of the sequencing read in order for the read to align, concordantly, to the wrong locus.
- The GTEx data that was analyzed relies on spliced alignment to the genome using STAR, and I presume the same tools were used to analyse the DGN cohort data (is this correct?). It would be enlightening to see if and how results would change if the reads were instead aligned using HISAT2. I am aware, for example, that HISAT2 takes special measures to avoid aligning reads to pseudogenes when similar quality alignments to transcripts of other biotypes are available (personal communication with Daehwan Kim). This may have some effect on the type and magnitude of false alignments you see (though, certainly, will not account for all of them).  Likewise, it would be very interesting to see how the analyses differ if alignment is done directly to the transcriptome using, e.g., Bowtie2 ( the authors already mention this possibility in the discussion section).
- I agree with Mike Love's suggestion that it seems important to explore the extent to which this effect may be mitigated by adopting more accurate methods for gene expression quantification.

I confirm that I have read this submission and believe that I have an appropriate level of expertise to confirm that it is of an acceptable scientific standard.

## References

[1] : Compressive mapping for next-generation sequencing. *Nature Biotechnology*.2016;34(4) : 10.1038/nbt.3511 374-376 10.1038/nbt.3511 PMC508083527054987

# Response

**Published:** 2019-04-09

**Authors:** Ashis Saha

**Affiliations:** Johns Hopkins University, USA

***Thank you for reviewing our paper.  Please see our responses inline in bold and italic font following your comments in standard font.***

 In this manuscript, Saha and Battle describe how errors in the genomic alignment of RNA-seq data can confound specific downstream analyses. Specifically, the focus on the discovery of trans-eQTLs and on co-expression analysis. Surprisingly, they find that, when a "naive" pipeline is used for trans-eQTL discovery, up to 75% of the trans-eQTL events detected may be false positives resulting from cross-mappability (the type of alignment error they discuss and characterize). In addition to describing this phenomenon, and demonstrating its effect on trans-eQTL and co-expression analysis, the authors also propose a new "cross-mappability" score, which allows one to map out which genes in a reference are likely to suffer from the types of spurious alignments, and subsequently, spurious correlations, that are described. The idea of cross-mappability seems a useful and logical extension of the mappability concept, where one is instead interested in which pairs or groups of genes share homologous sequence. The authors also provide pre-computed cross-mappability scores for hg19 and GRCh38.

 The paper is well-written, and the issues that the authors raise are important ones. It suggests that researchers should be cautious in interpreting the results of eQTL and co-expression analyses, and, importantly, provides them with tools to reassess their data and help control for the strong potential confounding factor of cross-mappability. I believe this is an important contribution. 

***>>  Thank you, we are appreciate your comments on our contribution.***

 My main questions and comments about the manuscript concern cross-mappability scores, and the alignment errors that lead to the observed problems.

 ●       Though the authors only explore the effect of alignment errors on eQTL and co-expression analysis, it seems that these types of issues could affect most analyses involving spliced-mapping of RNA-seq data to the reference genome. Specifically, the type of alignment errors illustrated in Figure 1 (A) would affect even basic expression quantification, let-alone co-expression analysis. This is particularly true for reads where this effect persists even when one considers only reads aligned uniquely by the tool. What would cause the aligner to return only a single (incorrect) locus for the read when multiple equally-good alignments should exist? Are the alignments contiguous at one locus but spliced at the other, or is the manner in which the read would align to the "true" and "spurious" locus different? Interestingly, it seems as though the cross-mappability map could act as a sort of homology table 1 that might even be useful for correcting these spurious alignments, or at least suggesting the true locus as an equally-good match.

***>>  Thank you for this comment. We agree that alignment errors potentially affect quantification of expression including splicing-aware quantifications. We observed cross-mapping errors even when only uniquely-mapped reads were used in the quantification (for e.g., in GTEx v7). There could be several reasons an aligner returns only a single incorrect locus for the read when multiple equally-good alignments should exist including genetic variation, errors in the reference genome, incorrect annotations, ambiguity due to splicing, and sequencing errors. It is difficult to assess the relative contribution of each of these.  We briefly mentioned the issues in the Introduction section of the manuscript.*** 

- ***"some alignment errors may remain between similar regions even among uniquely aligned reads due to genetic variation, errors in the reference genome, and other complications."***

 

***Regarding the pattern of alignment errors in "true" and "spurious" loci, we manually inspected suspected false positives, but did not observe any clear pattern of alignment errors. We observed multiple classes of errors including cases where alignment is contiguous at one locus (either true or spurious) but spliced at the other locus, and cases where alignment is contiguous at both loci.***

***Finally, it is a nice idea to see if alignment methods could directly utilize our cross-mappability resources or related approaches. We would be interested to follow up on this.***

 ●       Given that cross mappability is computed by mapping specific k-mers back to the genome (allowing up to 2 mismatches) using Bowtie, how does it deal with accounting for k-mers that span splicing junctions?  It seems to me that the specific case where reads map to pseudogenes rather than what is presumed to be the true (protein-coding) locus of the read could be explained by regions of the genome that are contiguous (un-spliced) in the pseudogene, but which span a splicing junction in the protein coding gene. If the cross-mappability score doesn't account for the cross-mappability of k-mer that may span splice junctions, then it seems it might miss such important cases. However, given that the score is computed assuming some known annotation, it would be possible to explicitly extract appropriately-sized contexts around each known splicing junction, and to include them into the reference that Bowtie maps against when computing the cross-mappability scores. How would the cross-mappability scores change if they also accounted for junction-spanning k-mers rather than just genomically contiguous k-mers? 

***>>  Thanks for raising this important issue. We did not account for k-mers spanning splice junctions and thus our current approach might miss some cross-mapping cases. However, we expect that for many genes with reasonable length exons, some k-mers that fall completely within the exon will also cross-map, and the genes will be identified as cross-mapping by our method in this case, but exceptions to this will also occur.  Cross-mappability scores would sometimes increase (but not decrease) if junction-spanning k-mers are considered, and we would need to consider whether these are also sometimes spurious. We mention in the manuscript that alignment to the transcriptome or splice-aware alignment might offer future improvements, but the computational cost and potential for some inaccuracies due to incorrect annotation would also have to be evaluated.***

***In future work, we  plan to extend cross-mappability scores using splice-aware alignments. One challenge here is to find all alignments of a k-mer using a spliced aligner. Spliced aligners are generally optimized to find the best alignment, and it is not as fast to find all alignments. Our current plan is to use STAR aligner (instead of Bowtie in the current setting) with a high number of multiple alignments allowed per read. In addition, we have to modify the k-mer generation process to include exon-exon junction spanning k-mers. Our plan is to evaluate every k-mer spanning exon-exon junctions of the annotated transcripts from a gene, along with the k-mers from the current collapsed gene model. As the k-mer generation process and the alignment process are going to be changed, we can no longer use the GEM Library to compute mappability of each k-mer; we will have to implement this part as well.  We leave the implementation of this, and evaluation, as future work, but agree it could offer improvements.***

 ●       Related to the above point, but thinking in the other direction, might the cross-mappability scores be too "conservative" in some cases? Specifically, the scores are computed using k-mers that are the length of relatively short reads for exons, and k-mers that are much shorter than typical reads for UTRs. However, the ambiguity of these sequences individually may not be sufficient to induce a false alignment when the reads being processed are paired-end reads.  In that case, one would expect misalignments to require that there be sequence ambiguity supporting both ends of the sequencing read in order for the read to align, concordantly, to the wrong locus.

***>>  This is a good observation. Yes, depending on the goal of the analysis, cross-mappability scores may be conservative in some situations. In a simple case, the value of k we used may be too short for some studies. One can easily recompute cross-mappabilities with appropriate k for the sequencing protocol, using our released code. Also, as you mentioned in the comment, misalignment of paired-end reads would require sequence ambiguity in both ends. Thus, our method may conservatively suggest two genes are cross-mappable, when paired-end reads would mostly or completely resolve the ambiguity. To compute cross-mappability incorporating pair-ended k-mers, we would need to model the distribution of fragment lengths in addition to splice junctions, which we have not yet addressed here. For simplicity and usability of our resources across different studies with different sequencing methods, we treat k-mers like single-ended reads. In general, small cross-mappability scores (where only a few k-mers overlap between genes) may not be sufficient to introduce alignment errors. One can easily filter gene pairs more stringently by thresholding the cross-mappability scores to higher numbers, which may weakly approximate requiring both ends of reads to align. Importantly, we do not claim that all instances of co-expression or trans-associations between cross-mappable pairs are actually false positives, some sequence similarity may occur between genes where a true hit also exists. One should consider the baseline rate of cross-mapping compared with that among observed hits, along with parameters of the study that may necessitate a specialized analysis.***

 ●       The GTEx data that was analyzed relies on spliced alignment to the genome using STAR, and I presume the same tools were used to analyse the DGN cohort data (is this correct?). It would be enlightening to see if and how results would change if the reads were instead aligned using HISAT2. I am aware, for example, that HISAT2 takes special measures to avoid aligning reads to pseudogenes when similar quality alignments to transcripts of other biotypes are available (personal communication with Daehwan Kim). This may have some effect on the type and magnitude of false alignments you see (though, certainly, will not account for all of them).  Likewise, it would be very interesting to see how the analyses differ if alignment is done directly to the transcriptome using, e.g., Bowtie2 ( the authors already mention this possibility in the discussion section).

***>>  Good points. Firstly, GTEx and DGN used different alignment and quantification tools. While GTEx v7 used STAR for alignment and RNA-SeQC for quantification, DGN used TopHat for alignment and HTSeq for quantification. False positives are observed for multiple tools. Secondly, we definitely agree avoiding pseudogenes (using --avoid-pseudogene option in HISAT2 or using other tools) would have an effect on the number of false positives. Another option is to simply not map trans-eQTLs for pseudogenes (or other biotypes) at all, which some studies in fact do.  As mentioned in the manuscript that 42.4% of eQTLs corresponding to protein-coding genes were also cross-mappable, compared to 75.14% of all eQTLs).  This is lower than for pseudogenes, but still above the expected background level, giving us an idea of the impact such a change would have. Of course, most researchers use public datasets without re-aligning them, so our resource shouldn’t rely on a specific aligner or parameters.  Finally, we agree that alignment to the transcriptome would be an important avenue for future research.***

 ●       I agree with Mike Love's suggestion that it seems important to explore the extent to which this effect may be mitigated by adopting more accurate methods for gene expression quantification.

***>>  We agree. We performed additional analyses using RSEM-quantified gene expressions and we still observed potential false positives in our analyses. Please see our response to Mike Love for details along with Supplementary Figure 11.***

# Peer review: Reviewer response for version 1

**DOI:** 10.5256/f1000research.18744.r41234

**Published:** 2018-12-12

**Authors:** Michael I. Love

**Affiliations:** 1 Department of Biostatistics, The University of North Carolina at Chapel Hill, Chapel Hill, NC, USA

The authors present a detailed analysis of how false positives arise in trans-eQTL and co-expression analyses, due to cross-mapping of reads among genes with sequence homology. They present a cross-mappability metric, and provide pre-computed cross-mappability for two human Gencode annotations (v19 and v26). They also provide software for efficiently computing cross-mappability available at a GitHub link. The command line software has detailed instructions online. The software requires genome FASTA files, a GTF file, a mappability bedgraph or bigwig file, Bowtie (v1) and an index, and a few R packages (data.table, intervals, argparser, stats).

 The report provides an important warning to the research community, and the pre-computed cross-mappability and the software will be a valuable resource for groups studying trans-eQTL and co-expression and making use of unique read counts for gene expression quantification.

 One of the key points of the article is noting that the cross-mappable and likely spurious trans-eQTLs are highly replicable between datasets, because " *it is driven by the underlying sequence of the genome, and similar alignment errors frequently occur regardless of tissue and study*." This logic also extends to co-expression and gene networks built on gene-gene expression correlations. Another key point was that filtering out of cross-mappable trans-eQTLs necessitates re-assessment of FDR, as the highly significant cross-mappable trans-eQTLs bring down the nominal FDR for other eQTLs.

 My main comment on the article is regarding details of the gene expression quantification.

 The authors note that, 

*"The number of reads misaligned to Gene B across samples may be directly proportional to the number of reads for Gene A, or may be determined by genetic variation creating sequence mismatches with the correct region....We note that such errors are not entirely mitigated by filtering multi-mapped reads—some alignment errors may remain between similar regions even among uniquely aligned reads due to genetic variation, errors in the reference genome, and other complications."*

 How would the analysis change if an expectation maximization (EM) approach were used for quantifying transcript and gene expression, where a latent variable is used for the origin of each read or pair of reads (both across isoforms and gene loci)? Such methods may resolve issues as shown in Figure 1A, because the observed coverage and expression of only gene A may give a higher likelihood than the observed coverage and expression of gene A and gene B. However the degree to which these methods may offer an improvement with respect to the cross-mappability issue depends on the distribution of genetic variation and errors in the reads, and on potential errors in the reference genome/transcriptome and on incomplete gene annotations. Therefore, it would be critical to perform the trans-eQTL analysis with regards to cross-mappability, when an EM algorithm, or a similar method that resolves multi-mapping reads, is used to quantify gene expression. Methods such as RSEM, Kallisto, or Salmon may be used to quantify gene and transcript abundance, which use EM or variational Bayes EM to resolve multi-mapping reads (as well as reads consistent with multiple isoforms of a gene) (disclosure: I am a co-author of the Salmon method).

 The authors note that:

*"Alignment to the transcriptome or splice-aware alignment may offer future improvements, but computational cost and inaccuracies due to incorrect annotation will have to be evaluated."*

 One potential solution which may alleviate both the issue of cross-mappability and incorrect annotation, would be to use an isoform discovery method to detect and characterize novel isoforms in a particular dataset (large datasets of rare tissues or sequencing of RNA from populations which have been under-represented in previous studies may very well discover novel isoforms), and then to use an EM or similar method to quantify expression.

 The details about how the GTEx (v7) and DGN data were quantified is missing, although these details are critical for understanding how broad the conclusions of the analysis may be. Gene expression was quantified in GTEx v7 using RNA-SeQC v1.1.8 which does not use an EM approach to resolve multi-mapping reads. According to the Methods section of Battle, et al (2014), for the DGN dataset, HTSeq was used to quantify gene expression, which also does not use an EM approach. I would recommend to add such quantification details of the datasets to this article.

I confirm that I have read this submission and believe that I have an appropriate level of expertise to confirm that it is of an acceptable scientific standard.

# Response

**Published:** 2019-04-09

**Authors:** Ashis Saha

**Affiliations:** Johns Hopkins University, USA

***Thank you for reviewing our paper.  Please see our responses inline in bold and italic font following your comments in standard font.***

 The authors present a detailed analysis of how false positives arise in trans-eQTL and co-expression analyses, due to cross-mapping of reads among genes with sequence homology. They present a cross-mappability metric, and provide pre-computed cross-mappability for two human Gencode annotations (v19 and v26). They also provide software for efficiently computing cross-mappability available at a GitHub link. The command line software has detailed instructions online. The software requires genome FASTA files, a GTF file, a mappability bedgraph or bigwig file, Bowtie (v1) and an index, and a few R packages (data.table, intervals, argparser, stats).

 The report provides an important warning to the research community, and the pre-computed cross-mappability and the software will be a valuable resource for groups studying trans-eQTL and co-expression and making use of unique read counts for gene expression quantification.

 One of the key points of the article is noting that the cross-mappable and likely spurious trans-eQTLs are highly replicable between datasets, because " *it is driven by the underlying sequence of the genome, and similar alignment errors frequently occur regardless of tissue and study*." This logic also extends to co-expression and gene networks built on gene-gene expression correlations. Another key point was that filtering out of cross-mappable trans-eQTLs necessitates re-assessment of FDR, as the highly significant cross-mappable trans-eQTLs bring down the nominal FDR for other eQTLs.

***>> Thank you for nicely summarizing the contribution of our work.***

 My main comment on the article is regarding details of the gene expression quantification.

 The authors note that, 

*"The number of reads misaligned to Gene B across samples may be directly proportional to the number of reads for Gene A, or may be determined by genetic variation creating sequence mismatches with the correct region....We note that such errors are not entirely mitigated by filtering multi-mapped reads—some alignment errors may remain between similar regions even among uniquely aligned reads due to genetic variation, errors in the reference genome, and other complications."*

 How would the analysis change if an expectation maximization (EM) approach were used for quantifying transcript and gene expression, where a latent variable is used for the origin of each read or pair of reads (both across isoforms and gene loci)? Such methods may resolve issues as shown in Figure 1A, because the observed coverage and expression of only gene A may give a higher likelihood than the observed coverage and expression of gene A and gene B. Howeverthe degree to which these methods may offer an improvement with respect to the cross-mappability issue depends on the distribution of genetic variation and errors in the reads, and on potential errors in the reference genome/transcriptome and on incomplete gene annotations. Therefore, it would be critical to perform the trans-eQTL analysis with regards to cross-mappability, when an EM algorithm, or a similar method that resolves multi-mapping reads, is used to quantify gene expression. Methods such as RSEM, Kallisto, or Salmon may be used to quantify gene and transcript abundance, which use EM or variational Bayes EM to resolve multi-mapping reads (as well as reads consistent with multiple isoforms of a gene) (disclosure: I am a co-author of the Salmon method).

***>> We appreciate the importance of this concern. We agree that EM-based quantification in principle has the potential to help address the cross-mappability issue to some extent.  We performed additional analyses using RSEM-quantified gene expressions and we still observed a high rate of potential false positives in our analyses, particularly for trans-eQTLs. We updated the manuscript along with a supplementary figure (Supplementary Figure 11).***

- ***"We also note that utilization of improved alignment and quantification methods to generate gene expression data may also be helpful to avoid false positives. For example, quantification of gene expression levels using RSEM[28], an expectation maximization based quantification tool, results in a smaller fraction of false positive trans-eQTLs (60.17%) than that using RNA-SeQC (75.14%). However, potential false positives due to cross-mappability still remain abundant in both trans-eQTL and co-expression studies (Supplementary Figure 11)."***

***We also added a brief description of the quantification pipelines of GTEx v7 and DGN in the revised manuscript.***

 The authors note that:

*"Alignment to the transcriptome or splice-aware alignment may offer future improvements, but computational cost and inaccuracies due to incorrect annotation will have to be evaluated."*

 One potential solution which may alleviate both the issue of cross-mappability and incorrect annotation, would be to use an isoform discovery method to detect and characterize novel isoforms in a particular dataset (large datasets of rare tissues or sequencing of RNA from populations which have been under-represented in previous studies may very well discover novel isoforms), and then to use an EM or similar method to quantify expression.

***>> This is an interesting idea.  For the current analysis, we plan to leave the approach as specified, but would like to explore this in the future.  We note, however, that EM-based approaches still do not address many cross-mappability errors in general, as shown in response to the previous question.***

 The details about how the GTEx (v7) and DGN data were quantified is missing, although these details are critical for understanding how broad the conclusions of the analysis may be. Gene expression was quantified in GTEx v7 using RNA-SeQC v1.1.8 which does not use an EM approach to resolve multi-mapping reads. According to the Methods section of Battle, et al (2014), for the DGN dataset, HTSeq was used to quantify gene expression, which also does not use an EM approach. I would recommend to add such quantification details of the datasets to this article.

***>>  We appreciate your concern. GTEx v7 used only uniquely mapped reads, but did not use an EM approach in the original analysis, though we have added the RSEM version for the revision as well. To make the manuscript self-contained, we briefly described the gene expression quantification pipeline of both GTEx v7 and DGN in the manuscript.***
